# Role of Intravitreal Bevacizumab in Management of Eale’s Disease

**DOI:** 10.12669/pjms.342.14483

**Published:** 2018

**Authors:** Mohammad Asim Mehboob, Muhammad Tahir, Huma Batool

**Affiliations:** 1 Dr. Mohammad Asim Mehboob, FCPS(Ophth), FICO, MRCSEd (Ophth). 69 Medical Battalion, Sialkot Cantt, Pakistan; 2 Dr. Muhammad Tahir, FCPS(Ophth). Headquarters Gilgit Baltistan Scouts, Gilgit; 3 Dr. Huma Batool, FCPS (Pulmonology) Assistant Professor. Department of Pulmonology, LGH Hospital, Lahore, Pakistan

**Keywords:** Bevacizumab, Retinal Vasculitis, Eale’s Disease

## Abstract

**Objective:**

To investigate the role of Intravitreal Bevacizumab (IVB), in preventing vitreo-retinal complications in patients of Eale’s Disease (ED).

**Methods:**

This randomized control trial was conducted at Armed Forces Institute of Ophthalmology (AFIO), Rawalpindi from May 2015 to December 2016. A total of 52 eyes of 26 patients, diagnosed with stage I or II of ED were randomly divided in two groups. Group A received monthly injections of IVB for 3 months, with steroids and laser photocoagulation. Group B received only steroids and laser treatment. Patients were followed for three months, and were analyzed for different clinical parameters.

**Results:**

Mean age of study population was 28.5±2.64 years. Difference in frequency of patients requiring PPV and showing regression in neovascularization was statistically significant between both groups (p=0.005 for both). However, difference in frequency of patients showing progression in stage of ED, regression of vasculitis and best corrected visual acuity at 12 weeks between two groups was not statistically significant (p= 0.012, 0.579, 0.046 respectively).

**Conclusion:**

Intravitreal Bevacizumab injection, given monthly in patients of ED results in significantly more regression in neovascularization, and less requirement for PPV, as compared to those receiving standard steroids and laser photocoagulation treatment.

## INTRODUCTION

Eale’s Disease (ED) is a commonly encountered idiopathic, inflammatory retinal vasculitis, affecting retinal veins of small and large caliber. It has been named after famous British ophthalmologist Henry Eales, who first described a series of young patients presenting with retinal vasculitis and visual deterioration.^1^ The etio-pathogenesis of ED is poorly understood, and remains debatable. Many studies have attributed the inflammation as response to Mycobacterium Tuberculosis antigens. The disease is very common in young adults, especially habitants of sub-continent.^2^ ED has overlapping stages of retinal vasculitis, non-perfusion, neovascularization, Vitreous Hemorrhage (VH) and Tractional Retinal Detachment (TRD). Most of the patients present with sudden visual loss due to VH, with bilateral and asymmetrical disease. If poorly managed, the disease can lead to permanent visual loss due to surgical complications, tractional or combined retinal detachment or neovascular glaucoma. Classification system for ED is also utilized to categorize the stage of disease and evaluate management options.^3^ ED can show spontaneous remission or relentless progression in different cases, and even different eyes of same patient. Appropriate and timely management can reduce significant visual mortality and morbidity attributed to the disease.^4^

Current treatment option includes corticosteroids, laser photocoagulation, retinal cryotherapy and surgical management in form of Pars Plana Vitrectomy (PPV) for VH or TRD. In the largest known study of ED, better outcome was reported for eyes treated with steroids and laser treatment. Also, late presentation was attributed to poor final outcome of disease.^5^ In a separate study, evaluation of vitreous fluid of patients of ED revealed increased expression of Vascular Endothelial Growth Factor (VEGF), highlighting the possible role of intravitreal anti-VEGF in treatment and managing progression of ED.^6^ Different case reports have reported efficacy of intravitreal anti VEGF injections in management of ED.^7^

The objective of this study was to investigate the role of Intravitreal Bevacizumab (IVB) injection, in preventing disease progression, regression of neovascularization and vasculitis, and need for PPV in patients of ED.

## METHODS

This randomized controlled trial was carried out at Armed Forces Institute of Ophthalmology, Rawalpindi, from May 2015 to December 2016, after approval from the institutional ethical review committee, and taking written informed consents from patients. A total of 56 eyes of 26 patients, diagnosed with ED on basis of classic retinal fundoscopic findings were analyzed. Patients from either gender, aged 20-40 years, with fundoscopic and laboratory evidence of ED, stage I to II of ED, with normal anterior segment examination and intraocular pressure were included. Patients with advanced ED, previous ocular surgery, glaucoma, diabetic retinopathy, stroke, hypertension, sickle cell disease, sarcoidosis, syphilis, rheumatoid arthritis, polyarteritis nodosa, oral ulcers, anterior uveitis, retinal detachment, high myopia, previous steroids use or laser photocoagulation were excluded. Sample size was calculated using World Health Organization sample size calculator keeping level of significance at 95% and power of test as 80%.^8^ Demographic data of study population was acquired. All patients underwent detailed ophthalmic examination with measurement of Best Corrected Visual Acuity (BCVA), anterior and posterior segment examination, measurement of intraocular pressure, peripheral fundus examination with Goldmann three mirror lens and ultrasonography, in cases of VH. Relevant hematological screening was done to rule out different infectious and non-infectious etiologies. All examination was done by single vitreo-retinal surgeon to exclude bias. Patients were divided in two groups by lottery method. Group A patients received three, monthly injections of IVB (Avastin, Genentech, USA) in dose of 1.25mg/0.05ml, through pars plana approach, 4mm from limbus using 25 gauge needle. Group B patients did not receive IVB. All patients received peri-ocular or intravitreal triamcinolone injection with peripheral laser photocoagulation and topical pressure lowering treatment. Those with positive tuberculin test also received systemic treatment of tuberculosis after consultation with internist. Patients were followed for 12 weeks, initially weekly for four weeks, and later on, at 8^th^ and 12^th^ week, with measurement of BCVA, intraocular pressure, anterior segment examination and fundoscopic examination for grading of ED and evaluation of response to treatment. Those requiring PPV were then referred to vitreo-retinal surgery department for surgical management of non-resolving VH or TRD. Data was entered in the pre devised proforma and confidentiality of the patient’s record was maintained. Statistical Package for Social Sciences (SPSS 20.0) for windows was used for statistical analysis. Descriptive statistics i.e. mean ± standard deviation for quantitative values (age, BCVA) and frequencies along with percentages for qualitative variables (gender, stage of ED, progression of stage, requirement of PPV, regression of neovascularization and vasculitis) were used to describe the data. We used Shapiro Wilk’s test to check normality of data. Qualitative variables were compared between two groups using Chi Square test and quantitative variables were compared using independent ‘t’ test. A p value of ≤0.005 was considered statistically significant.

## RESULTS

A total of 60 eyes of 30 patients were initially included. Four patients lost follow up during study. Finally, 52 eyes of 26 patients were analyzed. Mean age, gender distribution, requirement of PPV, stage progression, regression of neovascularization and vasculitis for study population and both groups is given in Table-I. There was no statistically significant difference between two groups in terms of age, gender, progression of disease and regression of vasculitis (p=0.414, 0.560, 0.012 and 0.576 respectively). In Group A, 9 (34.6%) eyes progressed to stage III or IV, while in Group B, 18 (69.2%) showed progression in severity of disease. Only 6 (23%) eyes in Group A and 16 (61.5%) eyes in Group B required PPV for non-resolving VH or TRD. Both groups showed comparable frequency of eyes showing regression of vasculitis (14 eyes in Group A and 12 in Group B). However, regression of neovascularization was observed more in Group A (16 eyes), as compared to Group B (6 eyes). The mean BCVA of both groups at 4 weeks, 8 weeks and 12 weeks, along with frequency of eyes with different stages of ED at presentation and 12 weeks is given in Table-II. There was no statistically significant difference between two groups for BCVA at presentation, four weeks and 12 weeks. However, there was statistically significant difference in BCVA of both groups at eight weeks (p=0.002). The difference in both groups in terms of final ED stage at 12 weeks was not statically significant (p=0.066). However, it is pertinent to mention that at 12 weeks, number of eyes in stage one was 15 (58%) in Group A as compared to 6 (23%) in Group B. Also, only 5 (19%) eyes progressed to stage IV in Group A, as compared to 12 (46%) in Group B. This shows considerably more number of eyes showing regression of ED in Group A, and less number of eyes showing progression to stage IV in Group A.

**Table-I:** Clinical data of study population (n=26).

Variable	Total n=26	Group A (IVB Group) n=13	Group B (No IVB Group) n=13	P Value (Between groups)
Age (Years) mean ± SD	28.5±2.64	28.08±3.08	28.92± 2.1	0.414*
Gender				0.560**
Male	17(65.4%)	9(69.2%)	8(61.5%)	
Female	9 (34.6%)	4(30.8%)	5(38.5%)	
PPV Required				0.005**
Yes	22(42.3%)	6(23.1%)	16(61.5%)	
No	30(57.7%)	20(76.9%)	10(38.5%)	
Stage Progression				0.012**
Yes	27(51.9%)	9(34.6%)	18(69.2%)	
No	25(48.1%)	17(65.4%)	8(30.8%)	
Regression in Neovascularization				0.005**
Yes	22(42.3%)	16(61.5%)	6(23.1%)	
No	30(57.7%)	10(38.5%)	20(76.9%)	
Regression in Vasculitis				0.579**
Yes	26(50%)	14(53.8%)	12(46.2%)	
No	26(50%)	12(46.2%)	14(53.8%)	

* Independent ‘t’ Test,

** Chi Square test.

**Table-II:** Comparison between two groups after IVB.

Variable		Group A (IVB Group) n=13	Group B (No IVB Group) n=13	P Value (Between Groups)
**BCVA (logMAR) mean±SD**				
Presentation		0.71±0.20	0.69±0.21	0.739*
4 weeks		0.73±0.16	0.79±0.13	0.155*
8 weeks		0.59±0.25	0.79±0.19	0.002*
12 weeks		0.62±0.28	0.78±0.29	0.046*
ED Stage – Number of eyes (%)				
Presentation	Ia	-	-	0.254**
	Ib	-	-	
	IIa	14 (53.8%)	18 (69.2%)	
	IIb	12 (46.2%)	8 (30.8%)	
12 weeks	Ia	10 (38.5%)	2 (7.7%)	0.066**
	Ib	5 (19.2%)	4 (15.4%)	
	IIa	2 (7.7%)	2 (7.7%)	
	IIb	-	-	
	IIIa	-	2 (7.7%)	
	IIIb	4 (15.4%)	4 (15.4%)	
	IVa	5 (19.2%)	12 (46.2%)	
	IVb	-	-	

* Independent ‘t Test,

** Chi Square test.

## DISCUSSION

It is believed that ED has inflammatory component leading to vascular occlusion and non-perfusion. Ischemic retina releases VEGF, which leads to neovascularization and fibrous element seen in fundi of ED patients. In a study conducted by Murugeswari P and associates, high angiogenic potential, and high pro-inflammatory factors were seen in vitreous samples of patients diagnosed with ED.^9^ This warrants use of IVB for managing the high angiogenesis which can have potential blinding complications. Use of IVB as an adjunct to other treatment modalities has been mentioned in literature.^4^ In another study, it was highlighted that excessive laser photocoagulation done conservatively for management of ED can result in iatrogenic retinal breaks formation, adding to the chances of retinal detachment.^10^ Use of IVB can spare excessive laser treatment, thus reducing number of visits and chances of retinal detachment.

ED can present with different clinical scenarios. Most common presentation is with VH, leading to sudden painless visual loss.^11^ However, another study has shown that ED does not have any common or typical presentation.^12^ We used IVB in patients presenting before VH or TRD. The reason for our methodology was to avoid unnecessarily causing proliferation of membranes, thus increasing chances of retinal breaks formation. Also, in presence of VH, one can never be sure, even after ultrasonography, for absence of membranes or tractions on retina. One author has advocated role of IVB, in releasing vitreoretinal traction and resolution of VH.^7^ Another study conducted on two patients of ED, with vitreoretinal traction and VH showed resolution of VH and traction. This study also showed no requirement of PPV, 6 months after IVB.^13^ One study recommends injection of bevacizumab before PPV, resulting in easy peeling of membranes and faster regression of neovascularization.^14^ Use of IVB in patients with VH and traction causes disadvantage of causing iatrogenic break formation. In another study, where IVB was used in patients of ED with dense VH, it was shown that IVB did not hasten resolution of VH, neither had it decreased the chances of undergoing PPV. They have shown that IVB can result in tractional retinal break formation, thus intravitreal injections are to be used with caution.^8^ In a study conducted on 14 eyes undergoing PPV for ED, it was observed that PPV has to be performed within seven days of IVB. Otherwise, the chances of TRD increase.^15^ We recommend that IVB be used in stage I or II, and has to be used with caution in stage III. Role of IVB in stage IV is recommended pre-operatively, for easy per-operative management of membranes and VH.

In our study, progression from earlier stage of ED was seen in 9 (34.6%) eyes in Group A, as compared to 18(69.2%) eyes in Group B. IVB thus helped in halting progression of the disease, though the results were not statistically significant. In a case report by Cp J et al, use of IVB in combination with laser photocoagulation resulted in stable BCVA and no progression in severity of ED stage.^16^ Since VEGF plays an important role in neovascularization, progression of ED severity can be monitored with use of IVB.

Our study has shown that significantly less number of patients required PPV after IVB injections, as compared to those not receiving it. This is contrary to study by Patwardhan SD et al, who have shown that in long term, IVB does not alter the requirement of PPV in patients of ED.^8^ The variation can be explained as the aforementioned study used IVB in patients with dense VH, and not in stage I or II. Our results are also advocated by Chanana B el al, who have shown that use of IVB may help in decreasing the requirement for PPV in patients with ED.^13^

Our study has shown that significant number of eyes showed regression in neovascularization in Group A, as compared to Group B. This is explained by reduction of angiogenic potential seen in ED patients by IVB. In a study by Kumar A et al, it was shown that monthly injections of IVB resulted in resolution of disc and retinal neovascularization in a patient of ED.^17^ Another study has shown the efficacy of IVB in resolution of neovascularization, where repeated laser treatment failed to regress fronds of retinal neovascularization.^18^ Our study did not find any difference in regression of vasculitis between two groups. This is obvious pertaining to absence of anti-inflammatory activity of bevacizumab.

Our study didn’t see significant difference between two groups at 12 weeks in terms of improvement in BCVA. In another study, role of ranibizumab was evaluated in a patient of macular edema due to ED. It was shown that ranibizumab only resulted in transient visual improvement, which later deteriorated.^19^ Role of IVB in patients of ED in terms of visual acuity is a debatable concept. Since visual acuity depends on multiple factors like involvement of macula, edema, cataract, tractional element threatening fovea and VH, improvement in BCVA cannot be taken as a yard stick to turn down the beneficial effects of bevacizumab in regression of neovascularization, requirement of PPV and progression in severity of ED stage.

### Limitation of this study

Small sample size, short follow up period and inability to evaluate the role of IVB in patients with VH but no tractional element. Further studies in this regard will help in finding a way forward in management of this potentially blinding condition.

## CONCLUSION

Intravitreal bevacizumab injection, given monthly in patients of ED stage I or II (hemorrhage, vasculitis, non-perfusion, neovascularization) results in significantly more regression in neovascularization, and less requirement for PPV, as compared to those receiving standard steroids and laser photocoagulation treatment. It must be considered as an adjunct to other treatment modalities for avoiding progression of disease and reducing requirement for surgical management of Eale’s disease.

### Authors’ Contribution

**MAM:** Conception and design of study, manuscript writing.

**MT:** Did data acquisition, data analysis.

**HB:** Did statistical analysis and manuscript editing.
